# Roles of plasminogen activator inhibitor-1 in aging-related muscle and bone loss in mice

**DOI:** 10.18632/aging.206318

**Published:** 2025-09-11

**Authors:** Takashi Ohira, Naoyuki Kawao, Yuya Mizukami, Kiyotaka Okada, Akihito Nishikawa, Hisatoshi Yamao, Ayaka Yamada, Hiroshi Kaji

**Affiliations:** 1 Department of Physiology and Regenerative Medicine, Kindai University Faculty of Medicine, Osaka 589-8511, Japan

**Keywords:** plasminogen activator inhibitor-1, aging, sarcopenia, osteoporosis, sex

## Abstract

Aging-related sarcopenia and osteoporosis are musculoskeletal disorders characterized by accelerated muscle and bone loss. Plasminogen activator inhibitor-1 (PAI-1), a fibrinolysis inhibitor, is involved in various pathological conditions, including sarcopenia and osteoporosis; however, its roles in aging-related sarcopenia and osteoporosis have yet to be fully investigated. Therefore, we investigated the roles of PAI-1 in aging-related sarcopenia and osteoporosis using PAI-1-gene-deficient and wild-type mice. Aging-related changes in muscle and bone were assessed by comparing the values in 24-month-old mice to those in 6-month-old mice. Regardless of sex, differences in muscle and bone parameters were observed between 24-month-old and 6-month-old mice. Aging increased PAI-1 expression in the gastrocnemius and soleus muscles of both female and male mice. PAI-1 deficiency significantly blunted aging-related decreases in lower limb muscle mass, muscle tissue weights, and grip strength in female mice but not in males. Moreover, PAI-1 deficiency significantly blunted aging-related cortical bone loss at the femurs and tibias of female but not male mice. These results indicate that PAI-1 is partly involved in aging-related sarcopenia and osteopenia in female mice, although the corresponding mechanisms remain unknown.

## INTRODUCTION

Sarcopenia, a disorder characterized by a decrease in skeletal muscle mass and strength, can be caused by various factors, including aging, [1, 2]. In aging-related sarcopenia, fast-twitch muscles are more susceptible to atrophy than slow-twitch muscles, with the pathophysiological changes in skeletal muscles increasing the risk for falls, fractures, and frailty and decreasing quality of life [2, 3]. In contrast, osteoporosis is a skeletal disorder characterized by a decrease in bone strength. Enhanced bone turnover and/or low mineralization decreases bone mineral density (BMD) and deteriorates the bone microarchitecture, consequently increasing the risk for fractures. Aging and sex play a crucial role in the pathophysiology of osteoporosis, with evidence suggesting an increase in the prevalence of osteoporosis with aging and menopause. The prevalence of aging-related sarcopenia also differs between males and females [4–7]. Given the increase in the elderly population worldwide, establishing countermeasures against aging-related sarcopenia and osteoporosis has become an urgent matter. The progression of aging-related sarcopenia involves multiple factors, such as neuromuscular junction degeneration, mitochondrial dysfunction, oxidative stress, and/or cellular senescence [3, 8]. Hence, a more detailed understanding of the mechanisms underlying sarcopenia and osteoporosis is needed to develop effective interventions for the prevention and treatment of these diseases.

Cellular senescence is induced when cells are exposed to various stressors, such as oncogene activation, mitochondrial dysfunction, and persistent DNA damage, which irreversibly suppresses cell proliferation. It has shown that cyclin-dependent kinase (Cdk) inhibitors, p21 (also known as Cdkn1a) and/or p16 (also known as Cdkn2a), are responsible for cell cycle arrest in cellular senescence [8–10]. Interventions targeting cellular senescence have the potential to prevent tumorigenesis and various diseases [11, 12]. Additionally, senescent cells secrete various proinflammatory cytokines and growth factors, a phenomenon called senescence-associated secretory phenotype (SASP), which contributes to aging-related diseases [8–10]. Previous studies have reported that the expression of p21 and p16 is upregulated in aged skeletal muscles of humans and mice; however, a considerable amount of evidence suggests that p16 has limitations as a marker of cellular senescence in mice [13, 14]. Cellular senescence in aged skeletal muscles may contribute to sarcopenia by inducing muscle stem cell dysfunction and SASP [8, 14]. Accumulating evidence has also suggested that cellular senescence plays a role in bone metabolism [15–17]. Taken together, these findings suggest that cellular senescence is crucially involved in the pathophysiology of sarcopenia and osteoporosis; however, the detailed mechanisms by which it does so have yet to be fully elucidated.

Plasminogen activator inhibitor-1 (PAI-1) has been shown to negatively regulate the fibrinolysis system by inhibiting plasmin production through the inactivation of plasminogen activators [18, 19]. Moreover, PAI-1 is known as a multifunctional factor involved in the regulation of cellular senescence [20, 21], apoptosis [22, 23], and inflammation [24, 25]. In fact, studies show that systemic and/or local increases in PAI-1 levels are involved in numerous pathological conditions, such as cancer, cardiovascular disease, diabetes mellitus, and muscle damage [18, 19]. Additionally, PAI-1 is one of the proteins produced in the SASP [26–29].

Our previous study in mice demonstrated that PAI-1 contributed to osteopenia and sarcopenia resulting from diabetes and glucocorticoid excess [18]. Moreover, a recent study showed that aging-related bone metabolic disruption was mitigated in PAI-1-deficient mice [30]. Genetic deletion or pharmacological inhibitors of PAI-1 have been found to partially suppress aging-related pathophysiological changes in humans and mice [31–33]. Khan et al. reported that heterozygous carriers of the null PAI-1 mutation had a longer life span compared to noncarriers of the null PAI-1 mutation, which appeared to be associated with a longer leukocyte telomere length, lower fasting insulin levels, and lower prevalence of diabetes mellitus [33]. Another study found that PAI-1 gene deficiency and oral administration of the PAI-1 inhibitor, TM5441, to Klotho gene-deficient mice impeded the progression of the aging-like phenotype by protecting organ structures and functions, thereby prolonging their life span [32]. Furthermore, Aihemaiti et al. showed that long-term administration of the small-molecule PAI-1 inhibitor, TM5484, prevented aging-related sarcopenia in male C57BL/6J mice [31]. They also found that oral TM5484 administration preserved the muscle fiber size and the isometric tension development of the gastrocnemius muscles in 12-month-old mice. Taken together, these findings suggest that PAI-1 might play a crucial role in aging-related sarcopenia and osteopenia; however, the underlying mechanisms have yet to be comprehensively elucidated.

The present study therefore examined the involvement of PAI-1 in aging-related sarcopenia and osteopenia using female and male PAI-1 gene-deficient (*PAI-1*^−/−^) and wild-type (*PAI-1*^+/+^) mice. We considered 6- and 24-month-old mice as young and old mice, respectively, and compared the aging-related phenotypes in the fast-twitch gastrocnemius and slow-twitch soleus muscles or femurs and tibias.

## RESULTS

### Effects of PAI-1 deficiency on aging-related decreases in muscle mass and grip strength in mice

**Figure 1:**
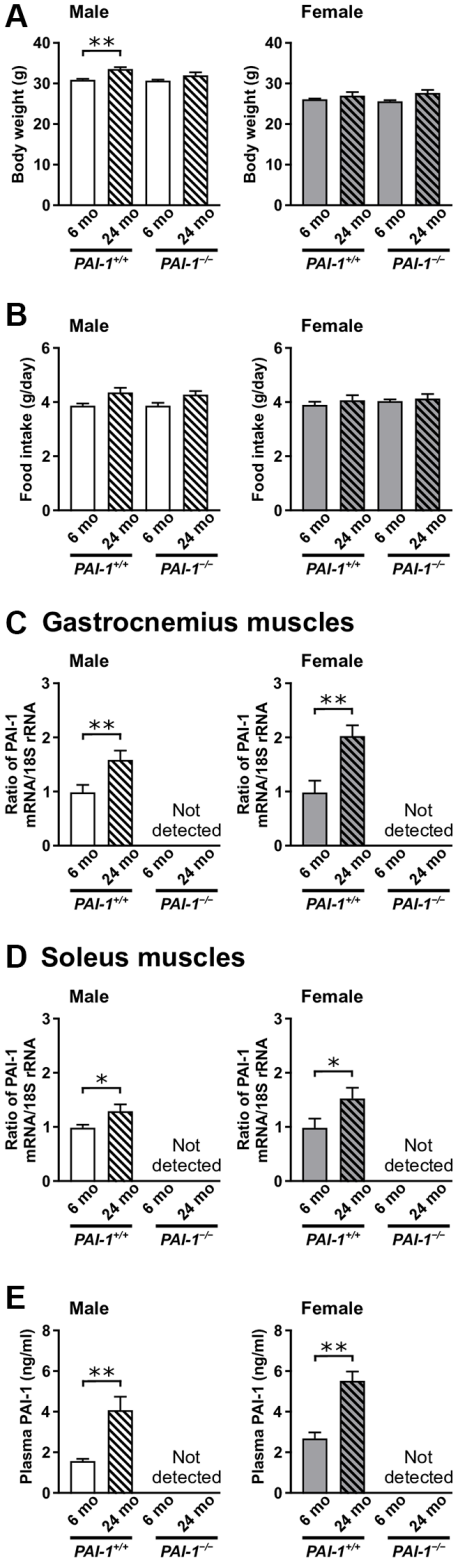
**Aging-related changes in body weight, food intake, and PAI-1 mRNA levels in the gastrocnemius and soleus muscles of male and female mice.** The figure shows the body weight (**A**), daily food intake (**B**), and PAI-1 mRNA levels in the gastrocnemius (**C**) and soleus (**D**) muscles of 6- and 24-month-old mice with PAI-1 gene deficiency (*PAI-1*^−/−^) and their wild-type counterparts (*PAI-1*^+/+^) (*n* = 9−10 in each group). Results of real-time PCR analysis for PAI-1 mRNA, expressed as a ratio to 18S rRNA, are shown. Plasma PAI-1 levels (**E**) in 6- and 24-month-old *PAI-1*^+/+^ and *PAI-1*^−/−^ mice are shown (*n* = 10 in each group). Data are presented as mean ± standard error of the mean. ^**^P < 0.01 and ^*^P < 0.05.

**Figure 2:**
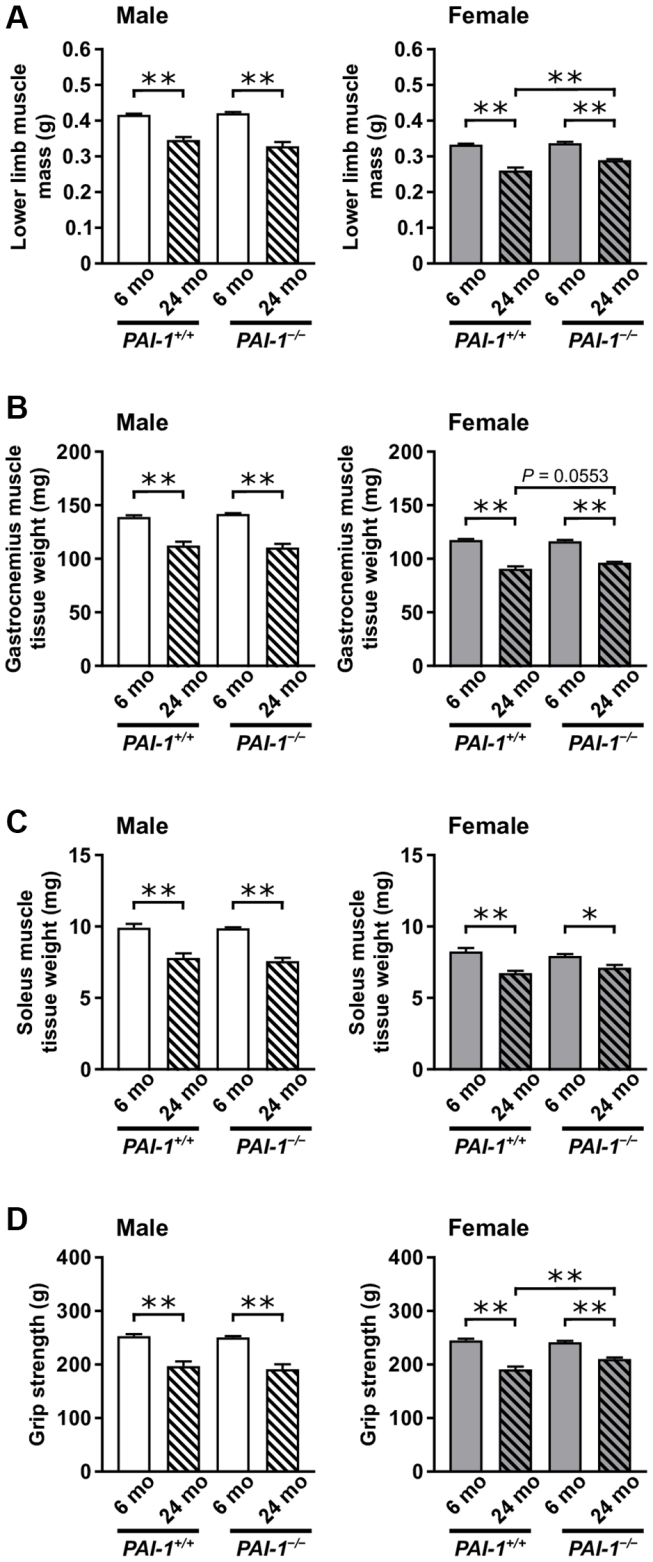
**Aging-related changes in muscle parameters in male and female mice.** Lower limb muscle mass assessed using qCT (**A**), tissue weights of the gastrocnemius (**B**) and soleus (**C**) muscles, and grip strength (**D**) in 6- and 24-month-old mice with PAI-1 gene deficiency (*PAI-1*^−/−^) and their wild-type counterparts (*PAI-1*^+/+^) are shown (*n* = 10 in each group). Data are presented as mean ± standard error of the mean. ^**^P < 0.01 and ^*^P < 0.05.

None of the mice died until the age of 6 months. The survival rates of 24-month-old male *PAI-1*^+/+^, female *PAI-1*^+/+^, male *PAI-1*^−/−^, and female *PAI-1*^−/−^ mice were 45%, 49%, 42%, and 75%, respectively. Body weights and daily food intakes were similar in 6- and 24-month-old *PAI-1*^+/+^ and *PAI-1*^−/−^ mice; however, 24-month-old male *PAI-1*^+/+^ mice had a significantly higher body weight than did 6-month-old male *PAI-1*^+/+^ mice (Figure 1A, 1B). PAI-1 mRNA levels increased significantly with aging in the gastrocnemius and soleus muscles of both male and female *PAI-1*^+/+^ mice (Figure 1C, 1D). Plasma PAI-1 levels increased significantly with aging in both male and female *PAI-1*^+/+^ mice (Figure 1E). Muscle mass of the lower limbs decreased significantly with aging in both male and female *PAI-1*^+/+^ mice (Figure 2A). Consistently, tissue weights of the gastrocnemius and soleus muscles significantly decreased with aging in both male and female *PAI-1*^+/+^ mice (Figure 2B, 2C). Moreover, grip strength decreased significantly with aging in both male and female *PAI-1*^+/+^ mice (Figure 2D). PAI-1 deficiency significantly blunted the decreases in both lower limb muscle mass and grip strength in 24-month-old female mice (Figure 2A, 2D). Aging-related changes in muscle parameters were evaluated as the ratio of values in 24-month-old mice to those in 6-month-old mice (Table 1). As shown in Table 1, PAI-1 deficiency significantly blunted aging-related changes in lower limb muscle mass, gastrocnemius and soleus muscle tissue weights, and grip strength in female but not male mice.

**Table 1:** Effects of PAI-1 deficiency on aging-related changes in muscle parameters of male and female mice.

	**Male mice**		**Female mice**	
	***PAI-1* ^+/+^**	***PAI-1* ^−/−^**	***PAI-1* ^+/+^**	***PAI-1* ^−/−^**
**Lower limb muscle mass**	−16.8 ± 2.2	−21.9 ± 3.0	−21.6 ± 2.7	−14.0 ± 0.8^*^
**Gastrocnemius muscle tissue weight**	−19.1 ± 2.8	−21.9 ± 2.6	−22.7 ± 2.2	−17.1 ± 0.7^*^
**Soleus muscle tissue weight**	−21.1 ± 3.3	−23.1 ± 2.6	−18.1 ± 2.1	−10.2 ± 2.6^*^
**Grip strength**	−21.9 ± 3.7	−23.4 ± 3.8	−21.8 ± 2.3	−13.0 ± 1.4^**^

Aging-related changes in lower limb muscle mass, gastrocnemius and soleus muscle tissue weights, and grip strength in mice with PAI-1 gene deficiency (*PAI-1*^−/−^) or wild-type mice (*PAI-1*^+/+^) are shown as the ratio of values for the 24-month-old mice to those for the 6-month-old mice. Data are presented as mean ± standard error of the mean, *n* = 10 in each group. ^**^P < 0.01 and ^*^P < 0.05 vs. *PAI-1*^+/+^ mice.

### Effects of PAI-1 deficiency on aging-related changes in the bone microarchitecture of mice

**Figure 3:**
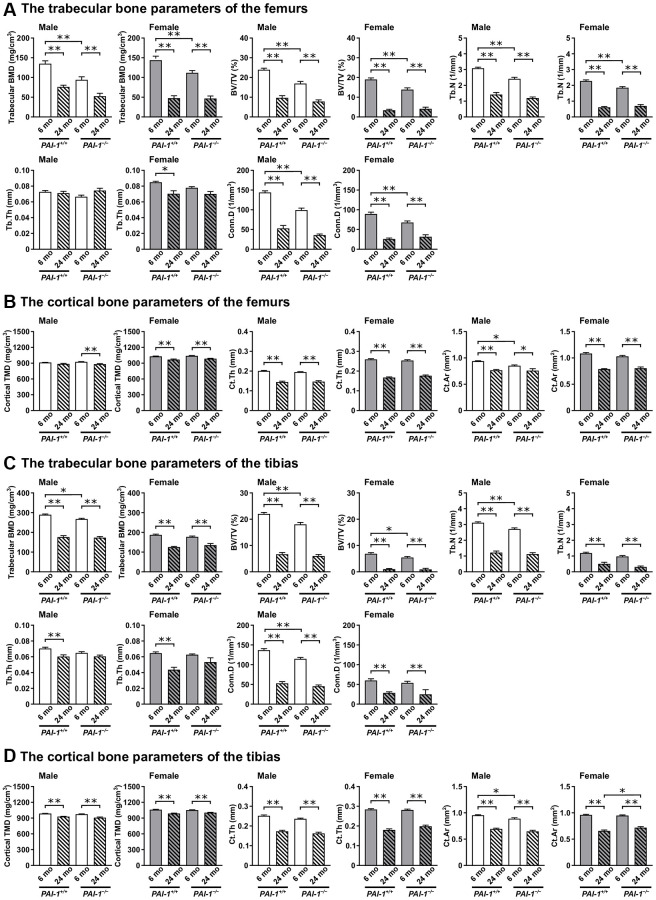
**Aging-related changes in the microarchitecture of long bones in male and female mice.** Trabecular and cortical bone parameters in the femurs (**A**, **B**) and tibias (**C**, **D**) of 6- and 24-month-old mice with PAI-1 gene deficiency (*PAI-1*^−/−^) and their wild-type counterparts (*PAI-1*^+/+^) are shown (*n* = 5−10 in each group). The distal metaphyseal regions of the femur and proximal metaphyseal regions of the tibia were analyzed using μCT. Trabecular bone parameters: trabecular bone mineral density (BMD), ratio of the segmented trabecular bone volume to the total tissue volume of the region of interest (BV/TV), trabecular number (Tb.N), trabecular thickness (Tb.Th), and connectivity density (Conn.D). Cortical bone parameters: cortical tissue mineral density (TMD), cortical thickness (Ct.Th), and cortical bone area (Ct.Ar). Data are presented as mean ± standard error of the mean. ^**^P < 0.01 and ^*^P < 0.05.

Trabecular BMD, the ratio of the segmented trabecular bone volume to the total tissue volume of the region of interest (BV/TV), trabecular number (Tb.N), connectivity density (Conn.D), cortical thickness (Ct.Th), and cortical bone area (Ct.Ar) in the femurs but not trabecular thickness (Tb.Th) and cortical tissue mineral density (TMD), significantly decreased with aging in male *PAI-1*^+/+^ mice (Figure 3A, 3B). Trabecular BMD, BV/TV, Tb.N, Tb.Th, Conn.D, cortical TMD, Ct.Th, and Ct.Ar in the tibias also significantly decreased with aging in male *PAI-1*^+/+^ mice (Figure 3C, 3D). Trabecular BMD, BV/TV, Tb.N, Tb.Th, Conn.D, cortical TMD, Ct.Th, and Ct.Ar in both the femurs and tibias significantly decreased with aging in female *PAI-1*^+/+^ mice (Figure 3A–3D). PAI-1 deficiency significantly blunted aging-related decreases in Conn.D, Ct.Th, and Ct.Ar in the femurs and cortical TMD, Ct.Th, and Ct.Ar in the tibias of female mice (Table 2A, 2B) and significantly prevented aging-related decreases in Tb.Th in the femurs of male mice (Table 2A).

**Table 2:** Effects of PAI-1 deficiency on aging-related changes in bone microarchitecture in the long bones of male and female mice.

**A**				
	**Male mice**		**Female mice**	
	***PAI-1* ^+/+^**	***PAI-1* ^−/−^**	***PAI-1* ^+/+^**	***PAI-1* ^−/−^**
**The trabecular bone parameters of the femurs**				
**Trabecular BMD**	−43.5 ± 3.4	−44.0 ± 8.1	−66.4 ± 4.6	−57.6 ± 5.9
**BV/TV**	−59.0 ± 4.9	−53.0 ± 5.0	−81.2 ± 2.8	−69.0 ± 6.2
**Tb.N**	−53.5 ± 4.5	−49.8 ± 2.9	−72.2 ± 2.4	−61.4 ± 4.9
**Tb.Th**	−2.2 ± 3.3	11.6 ± 4.6*	−17.1 ± 4.7	−10.0 ± 4.1
**Conn.D**	−62.8 ± 5.4	−63.2 ± 2.9	−70.2 ± 3.0	−52.5 ± 7.8^*^
**The cortical bone parameters of the femurs**				
**Cortical TMD**	−2.8 ± 1.1	−4.7 ± 1.1	−5.9 ± 1.1	−5.1 ± 0.6
**Ct.Th**	−27.6 ± 2.3	−24.6 ± 2.6	−35.1 ± 1.4	−30.1 ± 1.6^*^
**Ct.Ar**	−18.2 ± 1.5	−10.9 ± 4.6	−27.1 ± 0.9	−21.7 ± 2.5^*^

**B**				
	**Male mice**		**Female mice**	
	***PAI-1* ^+/+^**	***PAI-1* ^−/−^**	***PAI-1* ^+/+^**	***PAI-1* ^−/−^**
**The trabecular bone parameters of the tibias**				
**Trabecular BMD**	−38.9 ± 2.7	−34.9 ± 2.4	−32.7 ± 2.3	−23.4 ± 5.2
**BV/TV**	−69.2 ± 3.3	−66.0 ± 3.4	−83.0 ± 3.8	−80.5 ± 8.6
**Tb.N**	−60.2 ± 3.3	−57.9 ± 3.5	−56.0 ± 8.7	−66.3 ± 6.5
**Tb.Th**	−14.4 ± 3.2	−6.8 ± 2.7	−32.1 ± 5.0	−14.8 ± 9.5
**Conn.D**	−61.1 ± 3.5	−60.0 ± 3.0	−51.4 ± 4.6	−52.9 ± 24.2
**The cortical bone parameters of the tibias**				
**Cortical TMD**	−5.8 ± 0.6	−6.8 ± 1.3	−6.8 ± 0.7	−4.6 ± 0.7^*^
**Ct.Th**	−31.0 ± 2.1	−31.1 ± 2.9	−36.3 ± 2.1	−29.1 ± 2.1^*^
**Ct.Ar**	−27.0 ± 1.7	−26.9 ± 2.5	−31.4 ± 2.0	−24.0 ± 2.2^*^

Aging-related changes in the trabecular and cortical bone parameters in the femurs (**A**) and tibias (**B**) of mice with PAI-1 gene deficiency (*PAI-1*^−/−^) or wild-type mice (*PAI-1*^+/+^) are shown as the ratio of values for 24-month-old mice to those for 6-month-old mice. Trabecular bone parameters: trabecular bone mineral density (BMD), the ratio of the segmented trabecular bone volume to the total tissue volume of the region of interest (BV/TV), trabecular number (Tb.N), trabecular thickness (Tb.Th), and connectivity density (Conn.D). Cortical bone parameters: cortical tissue mineral density (TMD), cortical thickness (Ct.Th), and cortical bone area (Ct.Ar). Data are presented as mean ± standard error of the mean, *n* = 5−10 in each group. ^*^P < 0.05 vs. *PAI-1*^+/+^ mice.

### Effects of PAI-1 deficiency on muscle-related parameters in the gastrocnemius muscles of female mice

**Figure 4:**
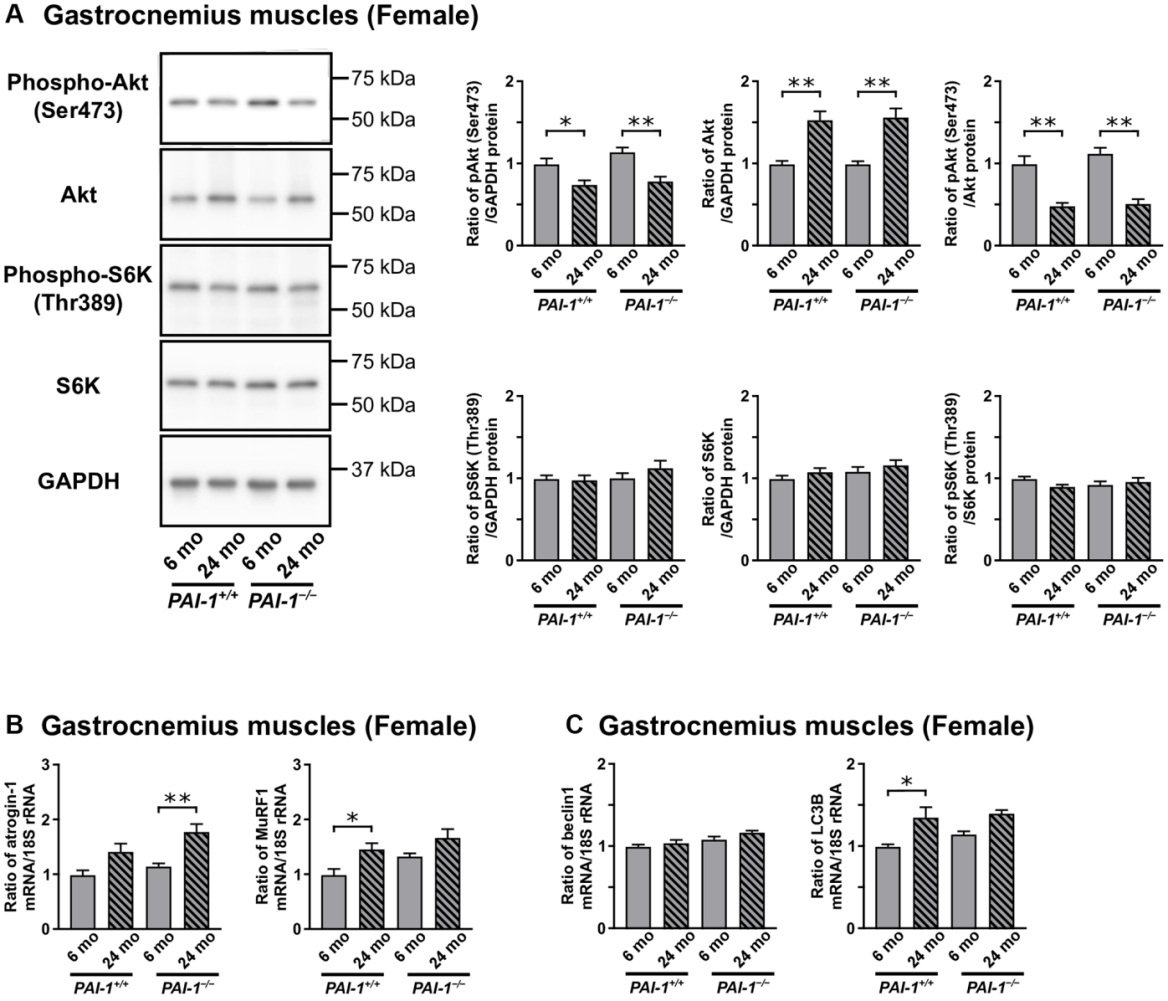
**Aging-related changes in gene or protein expression levels of protein synthesis and degradation markers in the gastrocnemius muscles of female mice.** Results of the Western blot analyses for components of the Akt/mechanistic target of the rapamycin (mTOR) pathway, Akt and p70S6 kinase (S6K), in the gastrocnemius muscles of 6- and 24-month-old female mice with PAI-1 gene deficiency (*PAI-1*^−/−^) and their wild-type counterparts (*PAI-1*^+/+^) (**A**) are shown (*n* = 9−10 in each group). The protein expression levels of phosphorylated Akt (Ser473), total Akt, phosphorylated S6K (Thr389), and total S6K are expressed as a ratio to GAPDH. Additionally, the phosphorylation rates of Akt (Ser473) and S6K (Thr389) are expressed as a ratio of phosphorylated to total protein levels. pAkt: phosphorylated Akt; pS6K: phosphorylated S6K. Results of real-time PCR analyses for the muscle-specific ubiquitin ligases (**B**), atrogin-1 and MuRF1, and autophagy-related genes (**C**), beclin1 and LC3B, in the gastrocnemius muscles of 6- and 24-month-old female *PAI-1*^+/+^ and *PAI-1*^−/−^ mice are shown (*n* = 9−10 in each group). The expression levels of atrogin-1, MuRF1, beclin1, and LC3B mRNA are expressed as a ratio to 18S rRNA. Data are presented as mean ± standard error of the mean. ^**^P < 0.01 and ^*^P < 0.05.

Muscle protein synthesis is mainly regulated by the Akt/mechanistic target of the rapamycin (mTOR) pathway [34], whereas muscle protein degradation is regulated by the ubiquitin–proteasome and autophagy–lysosome systems [34, 35]. Additionally, previous studies have shown that aging mainly affects fast-twitch (type II) muscle fiber-dominant muscles [2, 3]. Consistently, our results indicated that the effects of aging and/or PAI-1 deficiency on muscles were more pronounced in the gastrocnemius muscles of female mice. We therefore examined the effects of aging and/or PAI-1 deficiency on Akt/mTOR pathway activation and gene expression levels of muscle-specific ubiquitin ligases and autophagy-related factors in the gastrocnemius muscles of female mice. Accordingly, PAI-1 deficiency did not affect the protein levels of phosphorylated Akt (Ser473) or total Akt in the gastrocnemius muscles of female mice at either 6 or 24 months of age (Figure 4A). However, aging significantly decreased phosphorylated Akt (Ser473) levels and increased total Akt levels, which significantly decreased the phosphorylation rates of Akt (Ser473) in the gastrocnemius muscles of female mice (Figure 4A). However, the protein levels of total and phosphorylated (Thr389) S6K, a downstream component of the Akt/mTOR pathway, in the gastrocnemius muscles did not differ between 6- and 24-month-old female mice or between *PAI-1*^+/+^ and *PAI-1*^−/−^ mice (Figure 4A). Although the mRNA levels of ubiquitin ligases, atrogin-1 and MuRF1, appeared to increase with aging in the gastrocnemius muscles of female mice with or without PAI-1 deficiency, significant differences were only observed in atrogin-1 mRNA levels between 6- and 24-month-old female *PAI-1*^−/−^ mice and in MuRF1 mRNA levels between 6- and 24-month-old female *PAI-1*^+/+^ mice (Figure 4B). Additionally, no differences in the mRNA levels of autophagy-related genes, beclin1 and LC3B, were observed in the gastrocnemius muscles of *PAI-1*^+/+^ and *PAI-1*^−/−^ female mice; however, LC3B mRNA levels in these muscles significantly increased with aging in *PAI-1*^+/+^ mice (Figure 4C).

**Figure 5:**
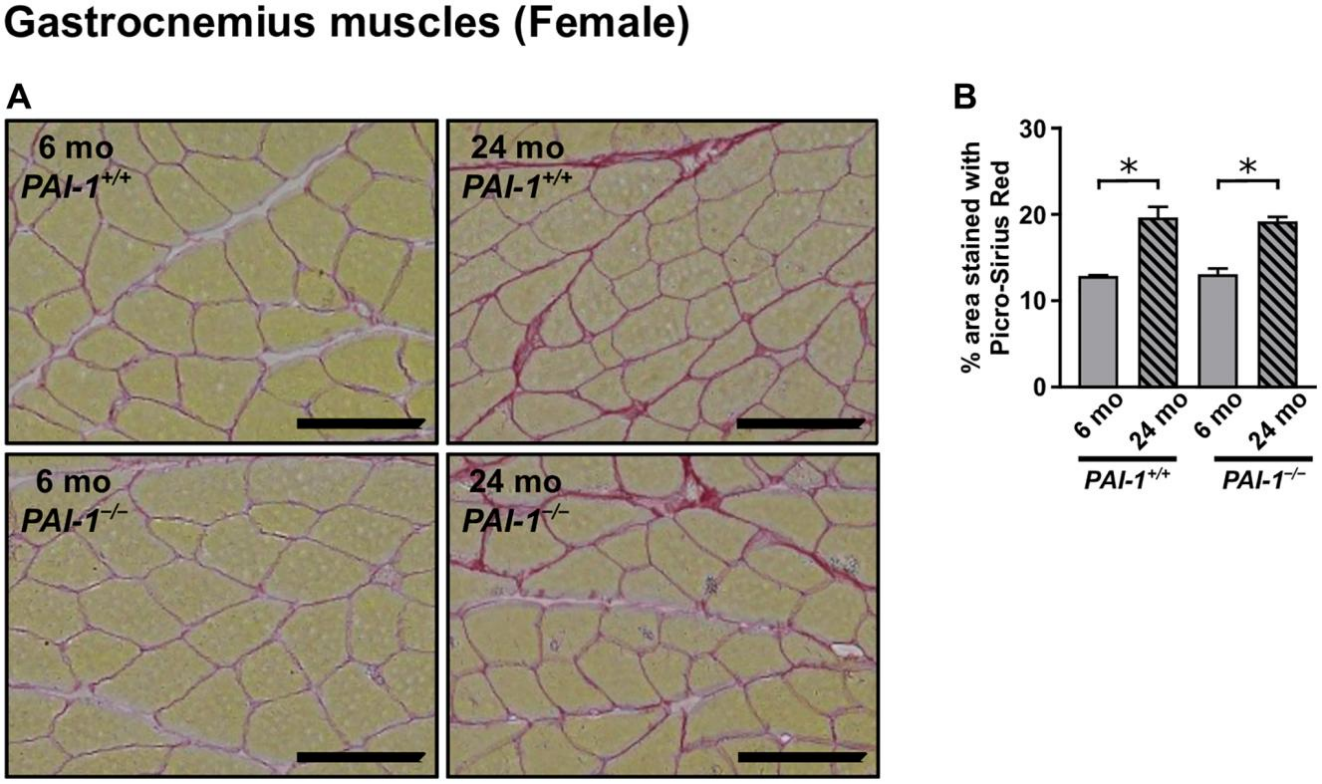
**Aging-related tissue fibrosis in the gastrocnemius muscles of female mice.** Picro-Sirius Red-stained cross-sections of the gastrocnemius muscles from 6- and 24-month-old female mice with PAI-1 gene deficiency (*PAI-1*^−/−^) and their wild-type counterparts (*PAI-1*^+/+^) are shown (**A**). Photomicrographs of four regions in each muscle section were analyzed, and the mean occupancy of collagen-containing fibrotic areas in tissue sections was calculated (**B**) (*n* = 3 in each group). Scale bars = 100 μm. Data are presented as mean ± standard error of the mean. ^*^P < 0.05.

Skeletal muscle tissue fibrosis affects muscle function and aging-related sarcopenia [36], we therefore examined the effects of aging and/or PAI-1 deficiency on muscle tissue fibrosis by performing Picro-Sirius Red staining on cross-sections of the gastrocnemius muscles from female mice. Notably, we found that aging significantly increased the fibrous tissue areas stained with Picro-Sirius Red in the gastrocnemius muscles of female mice, whereas PAI-1 deficiency had no effect on aging-related enhancement of fibrous tissue areas (Figure 5A, 5B).

### Effects of PAI-1 deficiency on the expression of cellular senescence markers in gastrocnemius muscles of female mice

**Figure 6:**
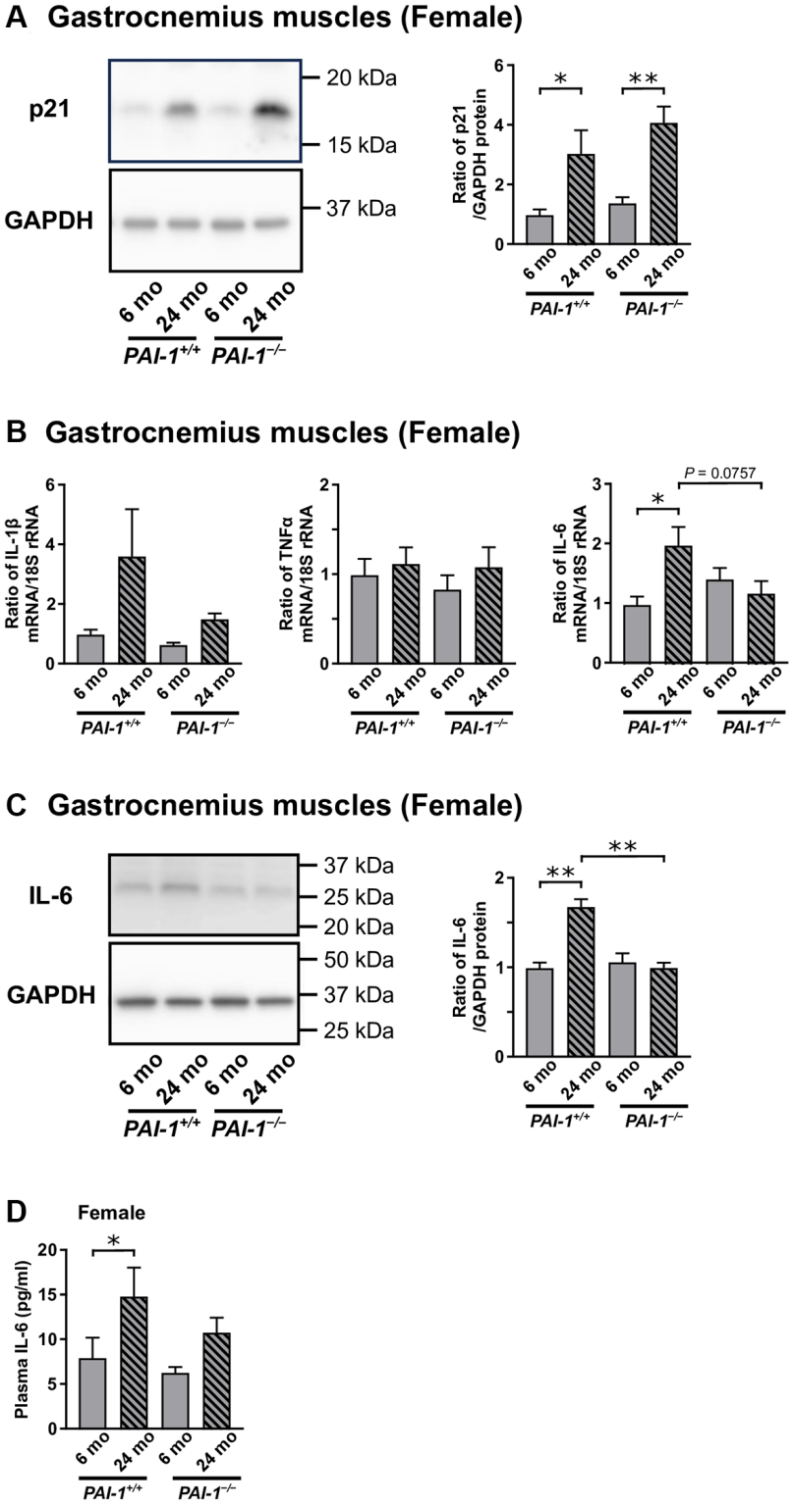
**Aging-related changes in the gene or protein expression levels of cellular senescence and senescence-associated secretory phenotype (SASP) markers in the gastrocnemius muscles of female mice.** The protein expression levels of the cellular senescence marker, p21, in the gastrocnemius muscles of 6- and 24-month-old female mice with PAI-1 gene deficiency (*PAI-1*^−/−^) and their wild-type counterparts (*PAI-1*^+/+^) are shown (**A**) (*n* = 8−10 in each group). Results of Western blot analysis for p21 are expressed as a ratio to GAPDH. Moreover, the gene expression levels of the SASP markers (**B**), IL-1β, TNFα, and IL-6, in the gastrocnemius muscles of 6- and 24-month-old female *PAI-1*^+/+^ and *PAI-1*^−/−^ mice are shown (*n* = 9−10 in each group). Results of real-time PCR analyses for IL-1β, TNFα, and IL-6 mRNA are expressed as a ratio to 18S rRNA. The protein expression levels of IL-6, expressed as a ratio to GAPDH, in the gastrocnemius muscles of 6- and 24-month-old *PAI-1*^+/+^ and *PAI-1*^−/−^ female mice are shown (**C**) (*n* = 4 in each group). Plasma IL-6 levels in 6- and 24-month-old *PAI-1*^+/+^ and *PAI-1*^−/−^ female mice are shown (**D**) (*n* = 9−10 in each group). Data are presented as mean ± standard error of the mean. ^*^P < 0.05.

Aging significantly increased p21 expression in the gastrocnemius muscles of female mice, regardless of PAI-1 deficiency (Figure 6A). We then examined the gene expression levels of SASP markers, IL-1β, TNFα, and IL-6 [8–10], in the gastrocnemius muscles of female mice (Figure 6B). Accordingly, aging significantly increased the mRNA levels of IL-6 but not IL-1β or TNFα in the gastrocnemius muscles of female *PAI-1*^+/+^ mice. PAI-1 deficiency tended to blunt the aging-related increase in IL-6 mRNA levels, with no significant difference between 24-month-old female *PAI-1*^+/+^ and *PAI-1*^−/−^ mice (*P* = 0.0757, Figure 6B). IL-6 protein levels increased significantly with aging in the gastrocnemius muscles of female *PAI-1*^+/+^ mice, whereas the aging-related increase in IL-6 protein levels was significantly suppressed in the muscles of 24-month-old female *PAI-1*^−/−^ mice (Figure 6C). Similarly, plasma IL-6 levels increased significantly with aging in female *PAI-1*^+/+^ mice (Figure 6D). PAI-1 deficiency tended to reduce the aging-related increase in plasma IL-6 levels in female mice, whereas no significant difference in plasma IL-6 levels was observed between female *PAI-1*^+/+^ and *PAI-1*^−/−^ mice at 24 months of age (Figure 6D).

## DISCUSSION

Aging has been found to induce sarcopenia and osteopenia in both sexes. The present study found that lower limb muscle mass, gastrocnemius and soleus muscle tissue weights, and grip strength were significantly lower in 24-month-old male and female wild-type mice than in their 6-month-old counterparts. Moreover, trabecular and cortical bone parameters in the femurs and tibias were significantly lower in 24-month-old male and female wild-type mice than in their 6-month-old counterparts. These results indicate that aging-induced both sarcopenia and osteopenia in mice. Furthermore, p21 levels in the gastrocnemius muscles were higher in 24-month-old female wild-type mice than that in their 6-month-old counterparts. These results indicate that the comparison between 6- and 24-month-old mice was appropriate for examining the effects of aging on skeletal muscles and bones.

A previous study found that the PAI-1 inhibitor, TM5484, effectively improved aging-related pathophysiological changes in the skeletal muscles of mice [31]. Furthermore, another study found that the PAI-1 inhibitor, PAI-039, exerted positive effects on the delayed repair of skeletal muscle injury in diabetic mice [37]. Additionally, our previous studies had demonstrated that PAI-1 was involved in glucocorticoid excess-induced sarcopenia in mice [38, 39], which suggests that PAI-1 negatively affects muscle mass and function. The results obtained herein revealed that aging significantly upregulated PAI-1 expression in the gastrocnemius and soleus muscles of both male and female mice. Moreover, PAI-1 deficiency significantly blunted aging-related loss in lower limb muscle mass, gastrocnemius and soleus muscle weights, and grip strength in females. These findings suggest that PAI-1 was partly involved in aging-related sarcopenia in female mice. Unfortunately, the present study was unable to identify PAI-I-producing cells in the skeletal muscles of mice; however, previous studies have suggested that PAI-1 was produced by vascular endothelial cells, fibroblasts, and macrophages rather than by myocytes in skeletal muscle tissues [26, 40, 41]. Therefore, vascular endothelial cells, fibroblasts, and monocytes/macrophages in muscle tissues may have contributed to the increase in PAI-1 mRNA levels in the skeletal muscles of mice observed in the present study.

The mechanisms by which PAI-1 facilitates aging-related sarcopenia in female mice remain unknown. The net balance between protein synthesis and degradation regulates skeletal muscle mass [34, 35]. However, the present study showed that regardless of PAI-1 deficiency, aging similarly affected parameters related to muscle protein synthesis, muscle protein degradation, and autophagy in the gastrocnemius muscles of female mice. Additionally, PAI-1 deficiency did not affect aging-induced fibrosis in the gastrocnemius muscles of female mice. Therefore, PAI-1 might not be associated with aging-related decrease in skeletal muscle mass and strength through muscle protein synthesis and degradation systems or muscle tissue fibrosis. Considerable evidence has shown that cellular senescence increases the expression levels of cell cycle inhibitors, such as p21, and SASP markers [8–10]. Similarly, the present study showed that aging upregulated p21 levels in the gastrocnemius muscles of female mice with or without PAI-1 deficiency, indicating the accumulation of senescent cells in these muscles with aging. However, we found that PAI-1 deficiency significantly attenuated the aging-related increase in the protein expression levels of IL-6, a SASP marker, in the gastrocnemius muscles of female mice. Additionally, although PAI-1 deficiency tended to blunt the aging-related increase in plasma IL-6 levels in female mice, no significant difference was observed between *PAI-1*^+/+^ and *PAI-1*^−/−^ female mice at 24 months of age. Similarly, PAI-1 deficiency suppressed increases in the plasma levels of IL-6 and IGFBP3 in Klotho-deficient mice [32]. These results suggest that PAI-1 could potentially mediate inflammation and positively regulate IL-6 expression in the gastrocnemius muscles of old female mice.

Extracellular IL-6 acts on cells via a cis- or trans-signaling mechanism, depending on whether the IL-6 receptors are membrane-bound or soluble, respectively, and activates the Janus kinase/signal transducer and activator of transcription pathway in cells [42, 43]. The functional roles of IL-6 in skeletal muscles are complex. In particular, acute exposure to IL-6 stimulates skeletal muscle growth, myogenesis, and energy production, whereas chronic IL-6 exposure results in skeletal muscle wasting [42–46]. Huang et al. demonstrated that the administration of tocilizumab, an anti-membrane-bound and soluble IL-6 receptor antibody, suppressed the increases in the expression of muscle-specific ubiquitin ligases and autophagy-related proteins induced by sciatic nerve transection in the tibialis anterior muscles, thereby preventing muscle atrophy in mice [44]. Meanwhile, Petersen et al. reported that IL-6 secreted by tumor cells accelerated the autophagic flux in skeletal muscle fibers via a trans-signaling mechanism, thereby potentially contributing to skeletal muscle wasting in patients with cancer cachexia [45]. Furthermore, Zanders et al. found that the skeletal muscle-specific deletion of the Il6st gene, which encodes an essential transmembrane protein (gp130) for the cis- and trans-signaling of IL-6, attenuated sepsis-induced muscle atrophy in mice [46]. Based on the available evidence, we can speculate that local increases in IL-6 levels in aged gastrocnemius muscles of female mice may have accelerated the progression of aging-related sarcopenia through PAI-1. Together with previous evidence suggesting that PAI-1 could be a SASP marker [26–29], SASP might be partly associated with aging-related sarcopenia through PAI-1. However, the present study showed that PAI-1 deficiency did not affect aging-related changes in the expression of markers of protein synthesis and degradation in the gastrocnemius muscles of female mice. Therefore, further extensive studies with different approaches are required to clarify the mechanism by which PAI-1 is involved in aging-related sarcopenia.

Recent studies have demonstrated that senescent cell depletion in bones blunted aging-induced osteopenia in mice, suggesting that cellular senescence and SASP are involved in the development of aging-related osteoporosis [15–17]. A considerable amount of evidence has shown that PAI-1 negatively regulates bone metabolism [18]. We had previously reported that PAI-1 plays a crucial role in the pathogenesis of osteopenia and delayed bone repair in mice with diabetes or glucocorticoid excess [39, 47–50]. Moreover, we had recently found that PAI-1 was associated with renal dysfunction-induced changes in the microstructure of the femoral trabecular bone in female but not male mice [51]. The present study found that PAI-1 deficiency blunted aging-related loss of cortical but not trabecular bone in female mice. Taken together, these findings suggest that aging-related cortical bone loss in female mice could occur partly through PAI-1. Our findings contradict the results presented in a previous study, which showed that PAI-1 deficiency significantly blunted aging-related trabecular bone loss in the femurs and lumbar spine of male mice [30]. However, the aforementioned study evaluated the influence of PAI-1 deficiency on aging-related osteopenia by comparing 6- and 18-month-old male mice. Accordingly, their findings revealed that PAI-1 deficiency decreased and increased trabecular bone parameters in the femurs and lumbar spine of 6- and 18-month-old male mice, respectively, suggesting differences in the effects of PAI-1 on trabecular bone between young and adult male mice. Furthermore, PAI-1 may play varying roles in cortical and trabecular osteopenia depending on the strain and age of the mice examined.

Our study revealed that PAI-1 deficiency partly blunted aging-related decreases in muscle mass, grip strength, and cortical bone parameters in female but not male mice, whereas aging similarly increased PAI-1 expression in the skeletal muscles of both male and female mice. We had previously reported on the presence of sex differences in the effects of PAI-1 deficiency on the musculoskeletal system of mice with diabetes [47, 52]. PAI-1 deficiency exacerbated the decrease in the grip strength of streptozotocin-injected female but not male mice [52]. PAI-1 deficiency suppressed streptozotocin-induced osteopenia in female but not male mice [47]. Additionally, *in vitro* experiments in the aforementioned study revealed that active PAI-1 treatment decreased osteogenic differentiation and mineralization in primary osteoblasts derived from female mice but not in osteoblasts derived from males [47]. Therefore, sex differences in the effects of PAI-1 deficiency may also be attributed to differences in responses between male and female cells to PAI-1, although the underlying mechanisms remain unknown. Considering that proteins encoded by genes on the sex chromosomes had been associated with sex differences in osteoporosis [53, 54], these proteins may also contribute to the differences in responses to PAI-1 in the muscles and bones of female and male mice. A study by Basurto et al. [55] showed that postmenopausal women had elevated circulating PAI-1 levels, presumably due to metabolic changes with aging rather than hormonal changes. We had previously reported that plasma PAI-1 levels and PAI-1 mRNA levels in the tibias were significantly higher in 14-week-old female mice than in their male counterparts, whereas PAI-1 mRNA levels in the gastrocnemius muscles were comparable between females and males [47]. These findings suggest that estrogen was not responsible for the aging-related increases in muscle PAI-1 expression, which were observed in the present study. Metabolic changes associated with aging may trigger the activation of PAI-1 gene transcription in various tissues, including skeletal muscles, in postmenopausal female mice [56]. Furthermore, aging-related metabolic changes in skeletal muscles of both humans and mice are dependent on sex [57, 58]. Therefore, the metabolic changes that occurred with menopause may have partly contributed to the sex differences in the effects of PAI-1 deficiency on muscle and bone observed in the present study. Further studies are certainly needed to clarify the mechanisms responsible for the sex differences in the role of PAI-1 in aging-related sarcopenia and osteopenia.

Several technical limitations of the current study warrant discussion. First, we cannot exclude the possibility that the characteristics of group-housed male mice may have masked the effects of PAI-1 deficiency on aging-related sarcopenia and osteopenia in male mice, resulting in the lack of significant differences having been observed. The larger standard error of the mean in the muscle parameters of 24-month-old male mice than in their female counterparts may be attributed to individual differences in food consumption and locomotor activity caused by intra-group ranking in group-housed male mice.

After comparing the muscle and bone parameters of 6- and 24- month-old *PAI-1*^+/+^ and *PAI-1*^−/−^ mice, we conclude that PAI-1 deficiency partly blunted aging-related muscle loss, decreased grip strength, and cortical bone loss in female mice. These results suggest that PAI-1 is partly involved in aging-related sarcopenia and cortical osteopenia in females. Nonetheless, further studies are needed to clarify the mechanisms of these actions of PAI-1.

## MATERIALS AND METHODS

### Animals

*PAI-1*^+/+^ and *PAI-1*^−/−^ mice that have a mixed genetic background of C57BL/6J (81.25%) and 129/SvJ (18.75%) were kindly provided by Professor D. Collen of the University of Leuven, Belgium [59]. Pairs of male and female mice of each strain were bred in a specific pathogen-free room with a 12/12-h light/dark cycle at the animal facility in Kindai University Faculty of Medicine. At the age of 4 weeks, male and female mice born from the parents of each strain were separated and three or four mice were housed in a cage (length: 15 cm, depth: 22 cm, height: 12 cm). Room temperature and humidity were maintained 20°C–24°C and 40–70%, respectively, with mice having *ad libitum* access to water and food (CE-2; Nihon CLEA, Tokyo, Japan). The CE-2 diet contained moisture (7.83%), crude protein (24.96%), crude fat (4.76%), crude fiber (4.84%), and crude ash (7.07%). The present study used 6- and 24-month-old mice to investigate the effects of PAI-1 deficiency on aging-related changes in skeletal muscle and bone. Prior to sample collection, the mice were individually caged for 2 weeks, and the daily food intake of each mouse was measured in the last week.

### Quantitative computed tomography (qCT)

Lower limb muscle mass of the mice was measured via qCT, as previously described [60, 61]. Regions from the proximal end to the distal end of the tibia were scanned to quantify lower limb muscle volume using an X-ray CT system *in vivo* (Latheta LCT-200; Hitachi Aloka Medical, Tokyo, Japan) and LaTheta software (version 3.41). The following formula was used to calculate muscle mass: Muscle mass (g) = Muscle volume (cm^3^) × Muscle density (1.06 g/cm^3^).

### Sample collection

Blood samples from 6- and 24-month-old mice were collected via cardiac puncture under isoflurane anesthesia. Blood clotting was inhibited by immediately mixing nine parts blood with one part 3.8% sodium citrate solution (FUSO Pharmaceutical Industries, Osaka, Japan). Plasma samples were then collected after centrifugation (1,500 × *g* at 4°C for 10 min). After euthanizing the mice via exsanguination under isoflurane anesthesia, the gastrocnemius and soleus muscles were isolated, measured for wet weights, and snap-frozen using liquid nitrogen. All samples were stored at −80°C until analyzed.

### Enzyme-linked immunosorbent assay (ELISA)

Plasma PAI-1 and IL-6 levels were assessed using a mouse total PAI-1 enzyme-linked immunosorbent assay kit (Cat. No. IMSPAI1KTT, Innovative Research, Novi, MI, USA) and a mouse IL-6 enzyme-linked immunosorbent assay kit (Cat. No. ELM-IL6-1, RayBiotech, Norcross, GA, USA), respectively. The procedures were conducted according to the manufacturers’ instructions.

### Real-time polymerase chain reaction (PCR)

Real-time PCR analysis was performed using an ABI StepOne Real-Time PCR System (Applied Biosystems, Foster city, CA, USA) with the Fast SYBR Green Master Mix (Applied Biosystems), as previously described [60, 61]. mRNA levels of the target genes were analyzed using the ΔΔCt method. Data were presented as normalized values with 18S ribosomal RNA (rRNA) levels.

The following real-time PCR primer sets were used in the analysis: PAI-1, forward 5′-TTCAGCCCTTGCTTGCCTC-3′, reverse 5′-ACACTTTTACTCCGAAGTCGGT-3′; IL-1β, forward 5′-GCCACCTTTTGACAGTGATGAG-3′, reverse 5′-GCTCTTGTTGATGTGCTGCT-3′; IL-6, forward 5′-GGATACCACTCCCAACAGACC-3′, reverse 5′-GCCATTGCACAACTCTTTTCTCA-3′; TNFα, forward 5′-ATGGCCTCCCTCTCATCAGT-3′, reverse 5′-CTTGGTGGTTTGCTACGACG-3′; Atrogin-1, forward 5′-GTCGCAGCCAAGAAGAGAAAGA-3′, reverse 5′-TGCTATCAGCTCCAACAGCCTT-3′; MuRF1, forward 5′-TAACTGCATCTCCATGCTGGTG-3′, reverse 5′-TGGCGTAGAGGGTGTCAAACTT-3′; Beclin1, forward 5′-TGAAATCAATGCTGCCTGGG-3′, reverse 5′-CCAGAACAGTATAACGGCAACTCC-3′; LC3B, forward 5′-CTGGTGAATGGGCACAGCATG-3′, reverse 5′-CGTCCGCTGGTAACATCCCTT-3′; 18S rRNA, forward 5′-CGGCTACCACATCCAAGGAA-3′, reverse 5′-GCTGGAATTACCGCGGCT-3′.

### Grip strength test

A grip strength meter (1027SM, Columbus Instruments, Columbus, OH, USA) was used to measure the grip strength of mice as previously described [51, 52, 60, 62]. Using its four limbs, the mice grasped the pull bar attachment, which was then pulled horizontally at a rate of ~2 cm/s. The maximum strength exerted while the mice grasped the bar was recorded. This test was performed five times for each mouse, and the average value was calculated.

### Micro-computed tomography (μCT)

The microarchitectures of the trabecular and cortical bones in the femurs and tibias of mice were assessed using μCT, as previously described [51, 62]. According to the guidelines of the American Society for Bone and Mineral Research [63], the distal metaphyseal segments of the femur and the proximal metaphyseal segments of the tibia in mice were scanned using CosmoScan GXII (Rigaku Corporation, Yamanashi, Japan). The microarchitecture of the femurs and tibias were analyzed using the software, Analyze 14.0 (AnalyzeDirect, Inc., Overland Park, KS, USA). To assess trabecular BMD, BV/TV, Tb.N, Tb.Th, and Conn.D of femurs and tibias, a 1-mm-thick region from the end of the growth plate to the diaphysis was set as the region of interest. To assess cortical TMD, Ct.Th, and Ct.Ar of femurs and tibias, a 1-mm-thick region in the mid-diaphysis was set as the region of interest.

### Western blot analysis

Western blot analyses were performed as previously described [60, 61]. The following primary antibodies obtained from Cell Signaling Technology were used for analyses: anti-phospho-Akt (Ser473) rabbit monoclonal antibody (4060S, 1:1000); anti-Akt rabbit monoclonal antibody (4691S, 1:1000); anti-phospho-p70S6 kinase (Thr389) rabbit monoclonal antibody (9234S, 1:1000); anti-p70S6 kinase rabbit monoclonal antibody (34475S, 1:1000); anti-IL-6 rabbit monoclonal antibody (12912S, 1:1000); anti-GAPDH rabbit monoclonal antibody (5174S, 1:10000). Anti-p21 rabbit monoclonal antibody (ab188224, 1:1000; Abcam, Cambridge, UK) was also used.

### Histological analysis

The right gastrocnemius muscles of female mice were embedded in optimum cutting temperature compound (Sakura Finetek Japan, Tokyo, Japan), immediately frozen in isopentane cooled using liquid nitrogen, and stored at −80°C. Muscle cross-sections (10-μm-thick) were then sliced in a cryostat (CM3050 S; Leica Biosystems, Nussloch, Germany). To assess muscle tissue fibrosis, the frozen sections were stained using the Picro-Sirius Red Stain Kit (For Cardiac Muscle, SRC-1; ScyTek Laboratories, Inc., PA, USA) according to the manufacturer’s instructions. Muscle sections were incubated in 0.2% phosphomolybdic acid solution for 5 min and rinsed in distilled water. Afterward, they were incubated in Picro-Sirius Red solution for 60 min and then rinsed in 0.5% acetic acid solution. Muscle sections were subsequently dehydrated in 99.5% ethanol (057-00451; FUJIFILM Corporation, Tokyo, Japan) and mounted in PARAmount-N (308-400-3; Falma, Inc., Tokyo, Japan). Images of Picro-Sirius Red-stained sections were obtained using a microscope (BZ-X710; Keyence, Osaka, Japan). Images of the four regions in each muscle section were analyzed using the BZ-X-Analyzer (Keyence), and the mean occupancy of collagen-containing fibrotic areas in the tissue sections was calculated in a blinded manner.

### Statistical analysis

All data were presented as mean ± standard error of the mean. Comparisons between four groups were performed using two-way analysis of variance and the Tukey–Kramer multiple comparison test, whereas comparisons between two groups were performed using an unpaired *t*-test. All statistical analyses were performed using GraphPad Prism version 7.02 software (GraphPad Software, La Jolla, CA, USA). In all analyses, *P*-values < 0.05 indicated statistical significance.
